# Association of Race/Ethnicity With Hospital Discharge Disposition After Elective Total Knee Arthroplasty

**DOI:** 10.1001/jamanetworkopen.2019.14259

**Published:** 2019-10-30

**Authors:** Jasvinder A. Singh, Michael J. Kallan, Yong Chen, Michael L. Parks, Said A. Ibrahim

**Affiliations:** 1 Medicine Service, Virginia Medical Center, Birmingham, Alabama; 2 School of Medicine, Department of Medicine, University of Alabama at Birmingham, Birmingham; 3 Department of Epidemiology, University of Alabama at Birmingham School of Public Health, Birmingham; 4 Perelman School of Medicine, Department of Biostatistics, Epidemiology and Informatics, University of Pennsylvania, Philadelphia; 5 Hospital for Special Surgery, New York, New York; 6 Department of Healthcare Policy and Research, Weill Cornell Medicine, New York-Presbyterian Hospital, New York, New York

## Abstract

**Importance:**

Total knee arthroplasty (TKA) is one of the most common elective procedures performed in adults with end-stage arthritis. Racial disparities in TKA outcomes have been described in the literature.

**Objectives:**

To assess the association of race/ethnicity with discharge disposition and hospital readmission after elective primary TKA and to assess the association of nonhome discharge disposition with hospital readmission risk.

**Design, Setting, and Participants:**

This retrospective cohort study used data from the Pennsylvania Health Care Cost Containment Council Database, a large regional database that included demographic data from all discharges of patients who underwent elective primary TKA in 170 nongovernmental acute care hospitals in Pennsylvania from April 1, 2012, to September 30, 2015. Data analyses were conducted from September 29, 2017, to November 29, 2017.

**Exposures:**

Patient race/ethnicity and discharge disposition.

**Main Outcomes and Measures:**

Discharge disposition and 90-day hospital readmission.

**Results:**

Among 107 768 patients, 7287 (6.8%) were African American, 68 372 (63.4%) were women, 46 420 (43.1%) were younger than 65 years, and 60 636 (56.3%) were insured by Medicare. In multivariable logistic regression, among patients younger than 65 years, African American patients were more likely than white patients to be discharged to inpatient rehabilitation facility (IRF) (adjusted relative risk ratio [aRRR], 2.49 [95% CI, 1.42-4.36]; *P* = .001) or a skilled nursing facility (SNF) (aRRR, 3.91 [95% CI, 2.17-7.06]; *P* < .001) and had higher odds of 90-day hospital readmission (adjusted odds ratio [aOR], 1.30 [95% CI, 1.02-1.67]; *P* = .04). Compared with white patients 65 years or older, African American patients 65 years or older were more likely to be discharged to SNF (aRRR, 3.30 [95% CI, 1.81-6.02]; *P* < .001). In both age groups, discharge to an IRF (age <65 years: aOR, 3.62 [95% CI, 2.33-5.64]; *P* < .001; age ≥65 years: aOR, 2.85 [95% CI, 2.25-3.61]; *P* < .001) or SNF (age <65 years: aOR, 1.91 [95% CI, 1.37-2.65]; *P* < .001; age ≥65 years: aOR, 1.55 [95% CI, 1.27-1.89]; *P* < .001) was associated with higher odds of 90-day readmission.

**Conclusions and Relevance:**

This cohort study found that race/ethnicity was associated with higher odds of discharge to an IRF or SNF for postoperative care after primary TKA. Among patients younger than 65 years, African American patients were more likely than white patients to be readmitted to the hospital within 90 days. Discharge to an IRF or SNF for postoperative care and rehabilitation was also associated with a higher risk of readmission to an acute care hospital.

## Key Points

**Question:**

Is there an association of patient race/ethnicity with discharge disposition or 90-day hospital readmission after elective primary total knee arthroplasty?

**Findings:**

In this statewide cohort study of 107 768 patients, African American patients were 2.5- to 5-fold more likely than white patients to be discharged to an inpatient rehabilitation facility or skilled nursing facility rather than home health care or home self-care. Among patients younger than 65 years, African American patients also had 1.3-fold higher odds of 90-day hospital readmission in people, but there was no difference in patients 65 years or older.

**Meaning:**

African American patients were associated with worse outcomes after primary total knee arthroplasty than white patients of the same age.

## Teaser

This cohort study examines the association of race/ethnicity with discharge disposition and 90-day hospital readmission overall and stratified by age among patients who have undergone total knee arthroplasty.

## Introduction

Total knee arthroplasty (TKA) is considered one of the most successful elective orthopedic procedures. Elective TKA is an effective treatment option for end-stage knee osteoarthritis (OA),^1,2^ an incurable condition that is rapidly increasing in prevalence and a leading cause of disability in elderly people.^3^ The utilization of joint replacement is projected to increase exponentially in the next decades.^4^ In national estimates, demand for TKA in the United States has been projected to increase by more than 600% from 2005 to 2030^5^ or by 400% from 2014 to 2040.^4^ In a 2009 national estimate,^6^ the number of total knee replacements performed in the United States exceeded 750 000 operations annually.

Although clinical indications for joint replacement, such as radiographic indications of knee OA-related^7,8,9,10^ or arthritis-related activity, work limitations, and severe pain, have been shown to disproportionately affect African American patients compared with white patients,^7,9,10,11,12,13,14^ a 2005 study^15^ reported marked racial variations in the receipt of elective joint replacement. The reasons for this disparity are complex and may involve patient-, clinician-, and system-level factors, including socioeconomic disparities, patient preference, medical comorbidity, depression, education, health literacy, and the patient-physician relationship.^16,17,18,19,20,21,22,23,24^ One aspect of the disparity may be whether there are variations in postoperative care and rehabilitation after the surgical procedure. In a 2015 analysis of patients who underwent TKA between 2008 and 2012,^25^ African American race/ethnicity was a significant risk factor for postoperative discharge destination even after adjusting for baseline comorbidity burden. In addition, discharge to an inpatient facility, such as an inpatient rehabilitation facility (IRF) or skilled nursing facility (SNF), was associated with increased odds of all-cause 30-day readmission to an acute care hospital.^25^

Since 2012, a number of Centers for Medicare & Medicaid Services policy experiments^26^ have occurred that incentivized better coordination of postoperative care and rehabilitation after elective joint replacement. One reason for ongoing interest in examining this issue is that a significant proportion of patients who undergo total joint replacement in the United States are institutionalized for postoperative care and rehabilitation.^25^ Furthermore, postoperative care and rehabilitation accounts for a significant portion of the overall cost of care per episode.^27^ In 2014, Centers for Medicare & Medicaid Services, the largest payer of total joint arthroplasty, introduced the Medicare Bundled Payment for Care Improvement initiative,^26^ which included payment models to hold hospitals accountable for Medicare costs related to lower extremity joint replacement for 90 days after patients’ hospital discharge.^26^

Whether these evolving policies have narrowed racial/ethnic variations in discharge destination remains unclear, to our knowledge. Therefore, the primary objectives of this analysis were to examine the racial/ethnic variation in discharge destination and 90-day hospital readmission after primary TKA. Our secondary objective was to assess whether postoperative discharge to an IRF or SNF after TKA was independently associated with higher odds of hospital readmission up to 90 days post-TKA, since 90-day hospital readmissions are not only costly and associated with increased morbidity burden, they also represent a major policy initiative for Medicare reimbursement.

## Methods

The institutional review board at the University of Pennsylvania approved this study as exempted from informed consent requirements because no individual person’s data were presented in any form in this database study. The study methods and results are described in accordance with the Strengthening the Reporting of Observational Studies in Epidemiology (STROBE) reporting guideline for observational studies.

### Study Sample

Our retrospective cohort study used the Pennsylvania Health Care Cost Containment Council (PHC4) Database, which includes statewide demographic characteristic data from all patient discharges from 170 nongovernmental acute care hospitals in the state of Pennsylvania. This includes all hospitals other than US Department of Veterans Affairs or military hospitals. As part of its enabling legislation, the PHC4 collects more than 5.2 million inpatient hospital discharge and ambulatory and outpatient procedure records annually from hospitals and freestanding ambulatory surgical centers throughout Pennsylvania. The data include hospital charges and treatment information that are collected on a quarterly basis and are subjected to standard validation processes by PHC4 and verified for accuracy by the facilities.^28^

The sample included all elective primary TKA performed in Pennsylvania from April 1, 2012, to September 30, 2015. The end date was chosen to correspond to the last day of the *International Classification of Diseases, Ninth Revision, Clinical Modification* (*ICD-9-CM*)^29^ coding system. We identified primary TKA by using the *ICD-9-CM* code 81.54. This study cohort and methodology has previously been described in detail.^30^

Only adults who identified as African American or white according to the PHC4 database and underwent an elective primary TKA were included in this study. Study exclusion criteria included patients with prior knee replacement; unknown gender, age, race/ethnicity or insurance status; bilateral knee replacement; death on the same day of the TKA surgical procedure or during hospitalization; transfer to a different acute care hospital or to a destination not included in our study; knee revision during the same hospitalization; missing metropolitan status for the facility; or a negative calculated readmission time from hospital discharge (ie, a likely administrative data set error).

### Study Outcome

The primary outcome of interest was discharge disposition after an inpatient elective TKA. This variable was categorized as home self-care (HSC), home health care (HHC), IRF, or SNF. Home self-care was used as the reference category. We also examined the risk of 30-, 60-, and 90-day acute care hospital readmissions.

### Risk Factors and Covariates

The primary risk factor of interest was patient race/ethnicity, categorized as white or African American. Patients with unknown or other race/ethnicity were excluded from the analyses. Covariates were chosen based on their associations with outcomes and complications after TKA or for being potential confounders.^30,31,32,33,34,35,36,37,38,39^ We adjusted for patient-level covariates, such as age, sex, insurance status (ie, private, Medicaid, Medicare or other government-sponsored health insurance program), and comorbidities, and facility-level variables, such as metropolitan area location and hospital annual TKA volume. Medical comorbidities were identified using the Quan-Charlson index^40^ and supplemented with the methods recommended by the Agency for Healthcare Research and Quality, versions 2.1 and 3.0 to 3.7^41^ (eTable 1 in the Supplement). Metropolitan area location was assessed using the 2013 US Department of Agriculture’s Rural-Urban Continuum Codes^42^ to assign the metropolitan area status to each hospital. The hospital TKA procedure volume was categorized into 3 levels based on the quarterly volume: (1) fewer than 50 TKA procedures per quarter, (2) 50 to 99 TKA procedures per quarter, and (3) 100 or more TKA procedures per quarter.

### Statistical Analysis

We tested the associations of race/ethnicity with the various patient-level, facility-level, and outcome variables using Wald χ^2^ from unadjusted binary or multinomial logistic regression models. All models considered race/ethnicity as the independent variable and accounted for clustering by the hospital facility.

Using similar strategies, patient-level characteristics, facility-level characteristics, and 30-, 60-, and 90-day hospital readmission were compared by postoperative discharge disposition (considered the independent variable). We also compared patient-level and facility-level characteristics along with postoperative discharge disposition by 30-, 60-, and 90-day hospital readmission status. Associations were tested using Wald χ^2^ from unadjusted binary or multinomial logistic regression models that also accounted for clustering by the hospital facility. Adjusted relative risk ratios (aRRRs) were calculated to assess the association of race/ethnicity with discharge disposition, with discharge to HSC as the reference category.

Unadjusted odds ratios (ORs) and adjusted ORs (aORs) of hospital readmission at 90 days were estimated using binary logistic regression models. Multivariable models were adjusted for patient-level and facility-level variables that were potentially associated with 90-day hospital readmission using a *P* value cutoff of less than .10 for inclusion in the multivariable model. In all models, patients were stratified by age group (<65 years vs ≥65 years). The age-based stratification accounted for differences in Medicare eligibility. These multivariable-adjusted models assessed the association of race/ethnicity with 90-day hospital readmission and the association of discharge disposition with 90-day hospital readmission by including all the patient and systems variables previously listed and also adjusting for discharge disposition.

Data management and analyses were conducted using SAS statistical software version 9.4 (SAS Institute) and Stata statistical software version 14.1 (StataCorp). *P* values were 2-tailed, and a *P* value of less than .05 was considered statistically significant for all results. Data analyses were conducted from September 29, 2017, to November 29, 2017.

## Results

### Demographic and Clinical Characteristics

There were 123 603 TKAs performed between April 1, 2012, and September 30, 2015, in Pennsylvania. We excluded 15 835 TKAs for meeting our exclusion criteria (eFigure 1 in the Supplement). The final analytic sample included 107 768 patients who underwent TKA (mean [SD] age, 66.2 [10.1] years; 68 372 [63.4%] women). There were 46 420 patients younger than 65 years (43.1%) and 61 348 patients 65 years or older (56.9%).

Among patients younger than 65 years, 4265 (9.2%) were African American and the rest were white; 223 African American patients (5.2%) and 1370 white patients (3.2%) were younger than 45 years. There were 3007 (70.5%) African American women and 25 467 (60.4%) white women younger than 65 years, and 1109 African American patients (26.0%) and 2612 white patients (6.2%) had Medicaid insurance (Table 1).

**Table 1.** Baseline Demographic and Clinical Characteristics by Race/Ethnicity and Age Group

Characteristic	Age <65 y			Age ≥65 y		
	No. (%)		*P* Value^a^	No. (%)		*P* Value^a^
	White (n = 42 155)	African American (n = 4265)		White (n = 58 326)	African American (n = 3022)	
Women	25 467 (60.4)	3007 (70.5)	<.001	37 575 (64.4)	2323 (76.9)	<.001
Age group, y						
<45	1370 (3.2)	223 (5.2)	<.001	NA	NA	<.001
45-54	10 471 (24.8)	1454 (34.1)		NA	NA	
55-64	30 314 (71.9)	2588 (60.7)		NA	NA	
65-74	NA	NA		35 551 (61.0)	2045 (67.7)	
75-84	NA	NA		19 760 (33.9)	869 (28.8)	
≥85	NA	NA		3015 (5.2)	108 (3.6)	
Insurance type						
Medicaid	2612 (6.2)	1109 (26.0)	<.001	73 (0.1)	36 (1.2)	<.001
Medicare or government	5774 (13.7)	1011 (23.7)		51 254 (87.9)	2597 (85.9)	
Private	33 769 (80.1)	2145 (50.3)		6999 (12.0)	389 (12.9)	
Facility-level metropolitan area	38 808 (92.1)	4224 (99.0)	<.001	53 615 (91.9)	3000 (99.3)	<.001
Volume of cases per quarter, No.						
<50	6927 (16.4)	806 (18.9)	.27	9193 (15.8)	560 (18.5)	.40
50-99	11 387 (27.0)	921 (21.6)		16 284 (27.9)	717 (23.7)	
≥100	23 841 (56.6)	2538 (59.5)		32 849 (56.3)	1745 (57.7)	
Complication						
Venous thromboembolism	260 (0.6)	40 (0.9)	.05	494 (0.8)	32 (1.1)	.30
Postoperative myocardial infarction	3 (<0.1)	0	NA	38 (0.1)	1 (<0.1)	.44
Prosthetic device complication	55 (0.1)	8 (0.2)	.48	35 (0.1)	1 (<0.1)	.56
Surgical wound infection	71 (0.2)	4 (0.1)	.21	118 (0.2)	0	NA
Comorbidity						
AIDS/HIV	6 (<0.1)	17 (0.4)	<.001	1 (<0.1)	3 (0.1)	.001
Blood loss anemia	320 (0.8)	36 (0.8)	.56	603 (1.0)	30 (1.0)	.86
Cardiac arrhythmias	3215 (7.6)	392 (9.2)	.02	9865 (16.9)	433 (14.3)	.002
Chronic pulmonary disease	6983 (16.6)	1087 (25.5)	<.001	8839 (15.2)	615 (20.4)	<.001
Coagulopathy	683 (1.6)	66 (1.5)	.79	1304 (2.2)	63 (2.1)	.70
Congestive heart failure	650 (1.5)	154 (3.6)	<.001	2345 (4.0)	198 (6.6)	<.001
Deficiency anemia	674 (1.6)	116 (2.7)	<.001	1194 (2.0)	105 (3.5)	.003
Depression	8960 (21.3)	690 (16.2)	<.001	7902 (13.5)	274 (9.1)	<.001
Diabetes, complicated	679 (1.6)	97 (2.3)	.03	1272 (2.2)	126 (4.2)	<.001
Diabetes, uncomplicated	7279 (17.3)	1002 (23.5)	<.001	12 359 (21.2)	974 (32.2)	<.001
Fluid and electrolyte disorders	2694 (6.4)	307 (7.2)	.35	5634 (9.7)	265 (8.8)	.40
Hypertension	24 291 (57.6)	2918 (68.4)	<.001	40 380 (69.2)	2328 (77.0)	<.001
Hypothyroidism	6619 (15.7)	358 (8.4)	<.001	12 202 (20.9)	371 (12.3)	<.001
Liver disease	669 (1.6)	116 (2.7)	<.001	525 (0.9)	51 (1.7)	<.001
Lymphoma	73 (0.2)	8 (0.2)	.82	160 (0.3)	13 (0.4)	.19
Metastatic cancer	22 (0.1)	5 (0.1)	.01	47 (0.1)	1 (<0.1)	.37
Nonbleeding peptic ulcer disease	160 (0.4)	17 (0.4)	.86	287 (0.5)	17 (0.6)	.54
Obesity	13 841 (32.8)	1890 (44.3)	<.001	12 492 (21.4)	1032 (34.1)	<.001
Other neurological disorders	1671 (4.0)	140 (3.3)	.04	2472 (4.2)	87 (2.9)	.002
Paralysis	112 (0.3)	17 (0.4)	.21	102 (0.2)	8 (0.3)	.27
Peripheral vascular disorders	476 (1.1)	54 (1.3)	.46	1848 (3.2)	95 (3.1)	.95
Psychoses	215 (0.5)	74 (1.7)	<.001	266 (0.5)	19 (0.6)	.16
Pulmonary circulation disorders	279 (0.7)	49 (1.1)	<.001	917 (1.6)	69 (2.3)	.02
Renal failure	881 (2.1)	220 (5.2)	<.001	4031 (6.9)	366 (12.1)	<.001
Rheumatoid arthritis or collagen vascular diseases	1785 (4.2)	235 (5.5)	.001	2406 (4.1)	155 (5.1)	.009
Solid tumor without metastasis	104 (0.2)	11 (0.3)	.87	347 (0.6)	15 (0.5)	.46
Substance use disorder	379 (0.9)	138 (3.2)	<.001	330 (0.6)	26 (0.9)	.046
Unhealthy alcohol use	550 (1.3)	94 (2.2)	<.001	354 (0.6)	20 (0.7)	.71
Valvular disease	869 (2.1)	82 (1.9)	.51	3269 (5.6)	135 (4.5)	.06
Weight loss	52 (0.1)	5 (0.1)	.92	104 (0.2)	5 (0.2)	.85

Abbreviation: NA, not applicable.

^a^ Using Wald χ^2^ from unadjusted logistic and multinomial models and accounting for the clustering by Pennsylvania area facility.

Among patients 65 years and older, 3022 patients (4.9%) were African American and the rest were white; 108 African American patients (3.6%) and 3015 white patients (5.2%) were 85 years or older. There were 2323 African American women (76.9%) and 37 575 white women (64.4%), and 36 African American patients (1.2%) and 73 white patients (0.1%) had Medicaid insurance (Table 1).

Table 2 summarizes demographic and clinical characteristics by discharge destination. Among 8382 people discharged to IRF, 602 (7.2%) were African American, 5879 (70.1%) were women, 6516 (77.7%) had Medicare insurance, and 3657 (43.6%) underwent TKA at high-volume hospitals. Among 23 170 people discharged to SNF, 2997 (12.9%) were African American, 17 126 (73.9%) were women, 16 893 (72.9%) had Medicare insurance, and 12 942 (55.9%) underwent TKA at high-volume hospitals. Among 52 672 patients discharged to HHC, 2744 (5.2%) were African American, 31 973 (60.7%) were women, 26 402 (50.1%) had Medicare insurance, and 29 663 (56.3%) underwent TKA at high-volume hospitals. Among 23 544 patients discharged to HSC, 944 (4.0%) were African American, 13 394 (56.9%) were women, 10 825 (46.0%) had Medicare insurance, and 14 711 (62.5%) underwent TKA at high-volume hospitals. eTable 2 and eTable 3 in the Supplement present these characteristics for African American patients and white patients, respectively.

**Table 2.** Demographic and Clinical Characteristics by Discharge Destination

Characteristic	Patients, No. (%)				*P* Value^a^
	Inpatient Rehabilitation Facility (n = 8382)	Skilled Nursing Facility (n = 23 170)	Home Health Care (n = 52 672)	Home Self-Care (n = 23 544)	
African American	602 (7.2)	2997 (12.9)	2744 (5.2)	944 (4.0)	<.001
Women	5879 (70.1)	17 126 (73.9)	31 973 (60.7)	13 394 (56.9)	<.001
Age group, y					
<45	52 (0.6)	128 (0.6)	932 (1.8)	481 (2.0)	<.001
45-54	466 (5.6)	1433 (6.2)	6810 (12.9)	3216 (13.7)	
55-64	1619 (19.3)	4537 (19.6)	18 090 (34.3)	8656 (36.8)	
65-74	2802 (33.4)	8047 (34.7)	18 626 (35.4)	8121 (34.5)	
75-84	2541 (30.3)	7589 (32.8)	7620 (14.5)	2879 (12.2)	
≥85	902 (10.8)	1436 (6.2)	594 (1.1)	191 (0.8)	
Insurance					
Medicaid	241 (2.9)	951 (4.1)	1909 (3.6)	729 (3.1)	<.001
Medicare or government	6516 (77.7)	16 893 (72.9)	26 402 (50.1)	10 825 (46.0)	
Private	1625 (19.4)	5326 (23.0)	24 361 (46.3)	11 990 (50.9)	
Facility-level metropolitan area	7217 (86.1)	21 952 (94.7)	49 589 (94.1)	20 889 (88.7)	<.001
Volume of cases per quarter. No.					
<50	2312 (27.6)	4264 (18.4)	8046 (15.3)	2864 (12.2)	<.001
50-99	2413 (28.8)	5964 (25.7)	14 963 (28.4)	5969 (25.4)	
≥100	3657 (43.6)	12 942 (55.9)	29 663 (56.3)	14 711 (62.5)	
Complication					
Venous thromboembolism	93 (1.1)	255 (1.1)	373 (0.7)	105 (0.4)	<.001
Postoperative myocardial infarction	13 (0.2)	16 (0.1)	9 (<0.1)	4 (<0.1)	<.001
Prosthetic device complication	12 (0.1)	15 (0.1)	54 (0.1)	18 (0.1)	.31
Surgical wound infection	25 (0.3)	61 (0.3)	74 (0.1)	33 (0.1)	<.001
Comorbidity					
AIDS/HIV	2 (<0.1)	9 (<0.1)	12 (<0.1)	4 (<0.1)	.39
Blood loss anemia	104 (1.2)	291 (1.3)	464 (0.9)	130 (0.6)	<.001
Cardiac arrhythmias	1478 (17.6)	4140 (17.9)	5832 (11.1)	2455 (10.4)	<.001
Chronic pulmonary disease	1635 (19.5)	4631 (20.0)	7947 (15.1)	3311 (14.1)	<.001
Coagulopathy	201 (2.4)	601 (2.6)	890 (1.7)	424 (1.8)	<.001
Congestive heart failure	480 (5.7)	1257 (5.4)	1150 (2.2)	460 (2.0)	<.001
Deficiency anemia	281 (3.4)	602 (2.6)	872 (1.7)	334 (1.4)	<.001
Depression	1528 (18.2)	4345 (18.8)	8464 (16.1)	3489 (14.8)	<.001
Diabetes, complicated	324 (3.9)	709 (3.1)	790 (1.5)	351 (1.5)	<.001
Diabetes, uncomplicated	2133 (25.4)	5790 (25.0)	9751 (18.5)	3940 (16.7)	<.001
Fluid and electrolyte disorders	870 (10.4)	2576 (11.1)	3744 (7.1)	1710 (7.3)	<.001
Hypertension (combined)	5715 (68.2)	16 065 (69.3)	33 576 (63.7)	14 561 (61.8)	<.001
Hypothyroidism	1922 (22.9)	4980 (21.5)	8922 (16.9)	3726 (15.8)	<.001
Liver disease	106 (1.3)	328 (1.4)	638 (1.2)	289 (1.2)	.59
Lymphoma	24 (0.3)	84 (0.4)	105 (0.2)	41 (0.2)	<.001
Metastatic cancer	10 (0.1)	24 (0.1)	28 (0.1)	13 (0.1)	.005
Nonbleeding peptic ulcer disease	38 (0.5)	138 (0.6)	213 (0.4)	92 (0.4)	.002
Obesity	2593 (30.9)	6887 (29.7)	13 662 (25.9)	6113 (26.0)	.002
Other neurological disorders	580 (6.9)	1261 (5.4)	1737 (3.3)	792 (3.4)	<.001
Paralysis	88 (1.0)	84 (0.4)	48 (0.1)	19 (0.1)	<.001
Peripheral vascular disorders	322 (3.8)	746 (3.2)	1059 (2.0)	346 (1.5)	<.001
Psychoses	86 (1.0)	258 (1.1)	166 (0.3)	64 (0.3)	<.001
Pulmonary circulation disorders	179 (2.1)	476 (2.1)	522 (1.0)	137 (0.6)	<.001
Renal failure	786 (9.4)	1942 (8.4)	1969 (3.7)	801 (3.4)	<.001
Rheumatoid arthritis or collagen vascular diseases	414 (4.9)	1172 (5.1)	2061 (3.9)	934 (4.0)	<.001
Solid tumor without metastasis	49 (0.6)	126 (0.5)	210 (0.4)	92 (0.4)	.006
Substance use disorder	88 (1.0)	262 (1.1)	383 (0.7)	140 (0.6)	<.001
Unhealthy alcohol use	67 (0.8)	268 (1.2)	480 (0.9)	203 (0.9)	.14
Valvular disease	475 (5.7)	1378 (5.9)	1820 (3.5)	682 (2.9)	<.001
Weight loss	19 (0.2)	74 (0.3)	58 (0.1)	15 (0.1)	<.001

^a^ Using Wald χ^2^ from unadjusted logistic and multinomial models and accounting for the clustering by Pennsylvania area facility.

### Discharge Destination by Race/Ethnicity

**Figure 1.**
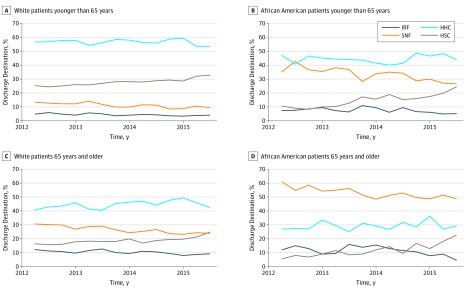
Rates Discharge Destination After Total Knee Arthroplasty Over Time Stratified by Patient Race/Ethnicity and Age Group HHC indicates home health care, HSC, home self-care; IRF, inpatient rehabilitation facility; and SNF, skilled nursing facility.

Figure 1 shows the association of discharge destination by race/ethnicity and age subgroup from 2012 to 2015. Among African American patients younger than 65 years, discharge to SNF decreased over time, while discharge to HSC increased over time. Among patients 65 years and older, the proportion of African American patients who were discharged to SNF decreased over time. However, almost 50% of African American patients were still discharged to SNF in 2015 (Figure 1).

### Discharge Destination by Race/Ethnicity From Multivariable Models

**Figure 2.**
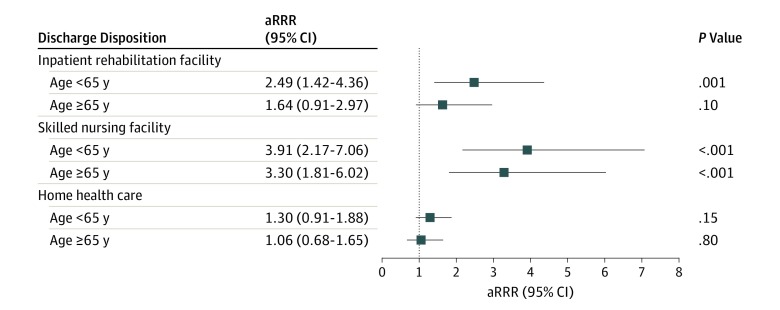
Association of African American Race/Ethnicity With Discharge Destination After Total Knee Arthroplasty Adjusted relative risk ratios (aRRRs) were calculated with white patients as the reference category and adjusted for age, gender, insurance type, comorbidities, metropolitan area location, hospital annual total knee arthroplasty volume, and comorbidities (except peptic ulcer disease, and solid tumor without metastasis in patients aged <65 years and prosthetic device complication, liver disease, metastatic cancer, solid tumor without metastasis, and unhealthy alcohol use in patients aged ≥65years because these variables had *P* values >.10). Squares indicate point estimates; bars, 95% CI.

Figure 2 shows the aRRRs of discharge destination by patient race/ethnicity. Among patients younger than 65 years, compared with white patients, African American patients were more likely to be discharged to an IRF (aRRR, 2.49 [95% CI, 1.42-4.36]; *P* = .001) or an SNF (aRRR, 3.91 [95% CI, 2.17-7.06]; *P* < .001) but not to HHC (aRRR, 1.30 [95% CI, 0.91-1.88]; *P* = .15). In the subgroup 65 years and older, compared with white patients, African American patients were more likely to be discharged to an SNF (aRRR, 3.30 [95% CI, 1.81-6.02]; *P* < .001) but not IRF or HHC.

### 90-Day Hospital Readmission by Race/Ethnicity From Multivariable Models

eFigure 2 in the Supplement presents the aORs of 90-day hospital readmission by patient race/ethnicity, adjusted for discharge disposition. Compared with white patients, African American patients younger than 65 years had higher odds of 90-day hospital readmission in people younger than 65 years (aOR, 1.30 [95% CI, 1.02-1.67]; *P* = .04). Race/ethnicity was not associated with 90-day hospital readmission in patients 65 years and older after adjusting for discharge disposition (aOR, 1.40 [95% CI, 0.96-2.06]).

### 90-Day Hospital Readmission by Discharge Disposition From Multivariable Models

**Figure 3.**
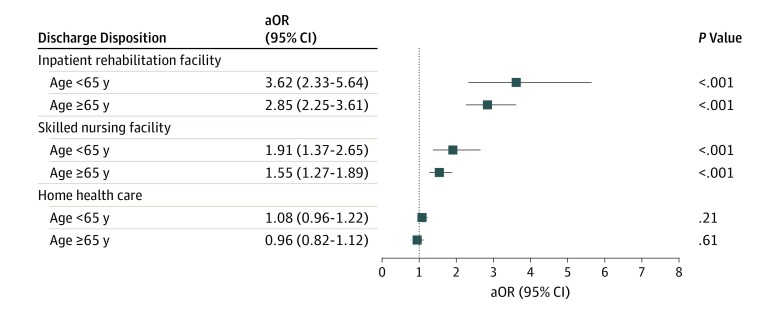
Association of Discharge Destination With Hospital Readmission Within 90 Days Stratified by Age Adjusted odds ratios (aORs) were calculated with home self-care as the reference category and adjusted for age, sex, insurance type, comorbidities, metropolitan area location, hospital annual total knee arthroplasty volume, and comorbidities (except for female sex, metropolitan area location, total knee arthroplasty volume by quarter, postoperative myocardial infarction, valvular disease, hypothyroid disease, and peptic ulcer disease in patients aged <65 years and total knee arthroplasty volume by quarter, prosthetic device complication, hypertension, hypothyroid disease, and solid tumor without metastasis in patients aged ≥65 years because these variables had *P* values >.10). Squares indicate point estimates; bars, 95% CI.

Figure 3 summarizes the odds of 90-day readmission to an acute care hospital by discharge disposition compared with HSC. In patients younger than 65 years, compared with patients discharged to HSC, patients had higher odds of 90-day readmission to acute care hospital if they had been discharged to IRF (aOR, 3.62 [95% CI, 2.33-5.64]; *P* < .001) or SNF (aOR, 1.91 [95% CI, 1.37-2.65]; *P* < .001); there was no association of 90-day hospital readmission with discharge to HHC (aOR, 1.08 [95% CI, 0.96-1.22]; *P* = .21). Similarly, in patients 65 years or older, compared with patients discharged to HSC, discharge to IRF (aOR, 2.85 [95% CI, 2.25-3.61]; *P* < .001) or SNF (aOR, 1.55 [95% CI, 1.27-1.89]; *P* < .001) was associated with higher odds of 90-day hospital readmission; there was no association of 90-day hospital readmission with discharge to HHC (aOR, 0.96 [95% CI, 0.82-1.12]; *P* = .61). Finally, sensitivity analyses using inverse probability weighting confirmed these findings with minimal accentuation of ORs but no change in significance or interpretation of findings.

## Discussion

In this cohort study using a large regional database, we found that there were significant variations in discharge disposition after elective TKA associated with a patient’s race/ethnicity. African American patients were significantly more likely to be discharged to IRFs or SNFs for postoperative care and rehabilitation compared with white patients with similar characteristics. This difference remained significant even after adjusting for confounders, such as demographic characteristics, comorbidity, and facility characteristics. Compared with discharge to HSC, discharge to IRF or SNF was associated with higher odds of 90-day hospital readmission for all patients regardless of age group. Among patients younger than 65 years, African American patients were more likely than white patients to be readmitted to the hospital within 90 days. These findings indicate that various Medicare policy innovations, including the Bundled Payment for Care Improvement^26^ for primary TKA (officially implemented in 2014, although many health care systems implemented the policy in anticipation), did not reduce racial/ethnic disparities associated with discharge disposition after TKA. Our analysis adds important information to the literature.

These findings are in keeping with previous studies that have examined this issue. A 2018 single-center study^43^ reported that 10% of patients were discharged to nonhome settings after TKA and that patients who were 75 years or older, were female, were not white, had Medicare insurance, had a history of depression, or had a high Charlson Comorbidity Index score were associated with higher risk for discharge to an IRF. A 2017 single-center study^44^ of 2869 patients who underwent TKA found that women, racial/ethnic minorities, and nonprivate insurance holders were more likely to be assigned to institutional care after discharge. In a 2016 analysis by Schwarzkopf et al^45^ of California state databases, race, age, insurance, and morbidity were significant factors associated with patient discharge destination. Schwarzkopf et al^45^ also found that compared with private health insurance, Medicare coverage was associated with discharge destination. A 2008 study by Hanchate et al^46^ suggested that limited insurance coverage and financial constraints may explain some of the racial/ethnic disparities in TKA utilization between African American patients and white patients, and these factors may have contributed to the differences noted in our study. Additionally, a 2015 analysis by Jorgenson et al^25^ found that compared with white patients, African American patients had significantly higher odds of discharge to an IRF or SNF.

Our findings that African American patients were more likely than white patients to be discharged to IRFs or SNFs for postoperative care and rehabilitation after elective TKA are important. Postoperative care and rehabilitation expenses are a source of marked cost variations per TKA episode.^27,47,48^ Also, discharge to an IRF or SNF is associated with higher odds of hospital readmission.^25^ It is possible that the decision on where to discharge patients after a surgical procedure is informed not only by clinical indications but also by social determinants of health,^49^ including socioeconomic status, employment, and social support, of which race/ethnicity might be a marker.^50^ Longer institutionalization after TKA among racial/ethnic minority patients may indirectly contribute to the concerns expressed by racial/ethnic minority patients regarding the safety and desirability of joint replacement as a treatment option.^51^ It is also possible that experiences of longer institutionalization after surgical procedures may be a factor in the well-documented lower preference among African American patients for joint replacement.^51^ We also noted relatively stable rates in our cohort of discharge to IRF after primary TKA over time, which suggests a stability in the utilization of this resource-intensive and costly rehabilitation treatment over time. This is consistent with previous studies,^52,53,54^ with similar or slightly lower rates of nonhome discharge disposition.

### Limitations and Strengths

There are important limitations to consider when interpreting the results of this study. First, this analysis used a large administrative database that was designed for hospital performance assessment but contains inadequate information on potential confounding variables, including body mass index, preoperative pain, preoperative function, radiographic indications of stage of underlying arthritis, preoperative or postoperative use of opioid medications, practice patterns, patient expectations, and hospital relationships with SNFs. Most of these potential confounders are associated with worse TKA outcomes and are more frequently encountered among African American patient populations,^31,55,56,57,58^ indicating that our study may have overestimated the association of discharge disposition with patient race/ethnicity. The database also had limited data on factors that may have mediated this association (ie, socioeconomic status, health literacy and beliefs, educational level, and postoperative complications).

 Second, our sample only included patients who underwent TKA and postacute rehabilitation in Pennsylvania. Our conclusions may not be readily extrapolated to other regions because there is significant regional variation regarding the rates of TKAs performed, with a 2- to 3-fold difference in TKA rates noted for Medicare beneficiaries in Pennsylvania among both white patients and racial/ethnic minorities.^18^

Third, patients receiving postoperative care and rehabilitation services may have used several different sites during an episode of care. The degree to which this potential crossover may have affected our findings is unknown yet must be considered in any interpretation of our results.

Fourth, discharge disposition and readmission rates can vary by setting (eg, single center, multicenter, region, state, national), state of residence, insurance status, study period, practice patterns, and patient characteristics (eg, age, income, education level, social support). While these might lead to differences in rates on a population or subpopulation level, it is unknown whether they would affect the disparities associated with race/ethnicity that we noted in TKA outcomes. Our study also had some strengths, including the use of statewide data from Pennsylvania and the adjustment for several known confounders of TKA outcomes.

## Conclusions

This cohort study of 107 768 patients who underwent primary TKA across 169 Pennsylvania hospitals found that African American patients were more likely to be referred to IRFs or SNFs for postoperative care and rehabilitation compared with white patients and that a post-TKA discharge to an IRF or SNF for rehabilitation was associated with greater odds of 90-day hospital readmission compared with being discharged to HHC or HSC. Future studies are needed to evaluate the decision-making process regarding discharge destination for postacute care and rehabilitation after elective TKA and how social determinants of health, such as patient race/ethnicity, affect these decisions. Future studies should also examine how changing Centers for Medicare & Medicaid Services policy reforms, such as Bundled Payment for Care Improvement, affect not only cost and quality of care but also equity.

## Notes

Supplement. **eTable 1.** Comorbidity Definitions and Corresponding *ICD-9-CM* Codes **eFigure 1.** Sample Flow Chart and Cohort Selection **eTable 2.** Demographic and Clinical Characteristics by Discharge Destination Among African-American Patients **eTable 3.** Demographic and Clinical Characteristics by Discharge Destination Among White Patients **eFigure 2.** Adjusted Association of Race/Ethnicity With 90-Day Hospital Readmission
