# Error in Author Affiliations

**DOI:** 10.1001/jamanetworkopen.2019.18528

**Published:** 2019-12-04

In the Original Investigation titled “Association of Race/Ethnicity With Hospital Discharge Disposition After Elective Total Knee Arthroplasty,”^1^ published October 30, 2019, there was an error in the first affiliation of Jasvinder A. Singh, MBBS, MPH. The affiliation should have read “Medicine Service, VA Medical Center, Birmingham, Alabama.” This article has been corrected.^1^
