# Supplementary material for: Association of Race/Ethnicity With Hospital Discharge Disposition After Elective Total Knee Arthroplasty
Source: JAMA Netw Open. 2019 Oct 30;2(10):e1914259. doi: 10.1001/jamanetworkopen.2019.14259 (PMC6824220; doi:10.1001/jamanetworkopen.2019.14259)
Supplement: Supplement. — eTable 1. Comorbidity Definitions and Corresponding ICD-9-CM Codes eFigure 1. Sample Flow Chart and Cohort Selection eTable 2. Demographic and Clinical Characteristics by Discharge Destination Among African-American Patients eTable 3. Demographic and Clinical Characteristics by Discharge Destination Among White Patients eFigure 2. Adjusted Association of Race/Ethnicity With 90-Day Hospital Readmission [file jamanetwopen-2-e1914259-s001.pdf]

Supplementary Online Content

Singh JA, Kallan MJ, Chen Y, Parks ML, Ibrahim SA. Association of race/ethnicity with hospital discharge disposition after elective total knee arthroplasty. *JAMA Netw Open*. 2019;2(10):e1914259. doi:10.1001/jamanetworkopen.2019.14259

eTable 1. Comorbidity Definitions and Corresponding *ICD-9-CM* Codes
eFigure 1. Sample Flow Chart and Cohort Selection
eTable 2. Demographic and Clinical Characteristics by Discharge Destination Among African-American Patients
eTable 3. Demographic and Clinical Characteristics by Discharge Destination Among White Patients
eFigure 2. Adjusted Association of Race/Ethnicity With 90-Day Hospital Readmission

This supplementary material has been provided by the authors to give readers additional information about their work.

© 2019 Singh JA et al. *JAMA Network Open.*

eTable 1. Comorbidity Definitions and Corresponding ICD-9-CM Codes

Elixhauser comorbidity	ICD-9-CM (Quan[41])	ICD-9-CM (Quan[41] + changes from AHRQ[42] V2.1, 3.0-3.7)
Congestive heart failure (CHF)	398.91, 402.01, 402.11, 402.91, 404.01, 404.03,<br>404.11, 404.13, 404.91, 404.93, 425.4-425.9, 428.x	No change
Cardiac arrhythmias	426.0, 426.13, 426.7, 426.9, 426.10, 426.12, 427.0-427.4, 427.6-427.9, 785.0, 996.01, 996.04, V45.0, V53.3	No change
Valvular disease	093.2, 394.x-397.x, 424.x, 746.3-746.6, V42.2, V43.3	No change
Pulmonary circulation disorders	415.0, 415.1, 416.x, 417.0, 417.8, 417.9	No change
Peripheral vascular disorders	093.0, 437.3, 440.x, 441.x, 443.1-443.9, 447.1,<br>557.1, 557.9, V43.4	Add: 442.0, 442.1, 442.2, 442.3, 442.81-<br>442.84, 442.89, 442.9, 449.x
Hypertension (combined)	401.x (uncomplicated),<br>402.x-405.x (complicated)	Add: 642.00, 642.01, 642.02, 642.03, 642.04 (uncomplicated),<br>401.0, 437.2, 642.10-642.11, 642.13-642.14, 642.20-642.24, 642.70-642.74,<br>642.90-642.94 (complicated)
Paralysis	334.1, 342.x, 343.x, 344.0-344.6, 344.9	Add: 438.20-438.22, 438.30-438.32, 438.40-438.42, 438.50-438.52, 780.72
Other neurological disorders	331.9, 332.0, 332.1, 333.4, 333.5, 333.92, 334.x-335.x, 336.2, 340.x, 341.x, 345.x, 348.1, 348.3, 780.3, 784.3	Add: 330.0-330.3, 330.8-330.9, 331.0, 331.11, 331.19, 331.2-331.4, 331.7, 331.81-331.82, 331.89, 333.7, 333.85, 333.94, 338.0, 347.00, 347.01, 347.10, 347.11, 649.30-649.34, 768.7, 780.31, 780.32, 780.97<br>Remove: 348.1, 348.3
Chronic pulmonary disease	416.8, 416.9, 490.x-505.x, 506.4, 508.1, 508.8	No change
Diabetes, uncomplicated	250.0-250.3	Add: 249.00, 249.01, 249.10, 249.11, 249.20, 249.21, 249.30, 249.31
Diabetes, complicated	250.4-250.9	Add: 249.40, 249.41, 249.50, 249.51, 249.60, 249.61, 249.70, 249.71, 249.80,<br>249.81, 249.90, 249.91
Hypothyroidism	240.9, 243.x, 244.x, 246.1, 246.8	No change
Renal failure	403.01, 403.11, 403.91, 404.02, 404.03, 404.13,<br>404.92, 404.93, 585.x, 586.x, 588.0, V42.0, V45.1, V56.x	No change
Liver disease	070.22, 070.23, 070.32, 070.33, 070.44, 070.54,<br>070.6, 070.9, 456.0-456.2, 570.x, 571.x, 572.2-<br>572.8, 573.3, 573.4, 573.8, 573.9, V42.7	Add: 573.9

© 2019 Singh JA et al. *JAMA Network Open.*

Peptic ulcer disease excluding bleeding	531.7, 531.9, 532.7, 532.9, 533.7, 533.9, 534.7, 534.9	Add: 531.41, 531.51, 531.61, 532.41, 532.51, 532.61, 533.41, 533.51, 533.61, 534.41, 534.51, 534.61
AIDS/HIV	042.x-044.x	No change
Lymphoma	200.x-202.x, 203.0, 238.6	Add: 203.12, 203.82
Metastatic cancer	196.x-199.x	Add: 209.70-209.75, 209.79, 789.51
Solid tumor without metastasis	140.x-172.x, 174.x-195.x	Add: 209.00-209.03, 209.10-209.17, 209.20-209.27, 209.29, 209.30-209.36, 258.01-258.03
Rheumatoid arthritis / collagen vascular diseases	446.x, 701.0, 710.0-710.4, 710.8, 710.9, 711.2, 714.x, 719.3, 720.x, 725.x, 728.5, 728.89, 729.30	No change
Coagulopathy	286.x, 287.1, 287.3-287.5	Add: 289.84, 649.30-649.34
Obesity	278.0	Add: V85.3, V85.4, 649.10-649.14, 793.91, V85.54
Weight loss	260.x-263.x, 783.2, 799.4	No change
Fluid and electrolyte disorders	253.6, 276.x	No change
Blood loss anemia	280.0	Add: 648.20-648.24
Deficiency anemia	280.1-280.9, 281.x	Add: 285.21, 285.22, 285.29
Alcohol abuse	265.2, 291.1-291.3, 291.5-291.9, 303.0, 303.9, 305.0, 357.5, 425.5, 535.3, 571.0-571.3, 980.x, V11.3	Add: 291.0<br>Remove: V11.3
Drug abuse	292.x, 304.x, 305.2-305.9, V65.42	Add: 648.30-648.34
Psychoses	293.8, 295.x, 296.04, 296.14, 296.44, 296.54, 297.x, 298.x	No change
Depression	296.2, 296.3, 296.5, 300.4, 309.x, 311	No change

© 2019 Singh JA et al. *JAMA Network Open.*

eFigure 1. Sample Flow Chart and Cohort Selection

n = 123,603

All knee replacements performed from 2012Q2 through 2015Q3 (4/1/2012-9/30/2015)

↓

Exclude: n = 15,835

1. Race missing or not listed as African American or white (n=4,722)
2. Age <18 years (n=16)
3. Missing/unknown gender (n=0)
4. Those with bilateral knee replacement [of which one was considered the index surgery] (n=9,777)
5. Died on the same day as [index surgery] knee replacement (n=1)
6. Died in hospital [no other limitations] following knee replacement (n=56)
7. Transferred out (n=204)
8. Went to a non-study destination (n=242)
9. Had knee revision during the same hospitalization (n=154)
10. Had hip replacement and/or revision during the same hospitalization (n=24)
11. More than two previous knee replacements during period (n=12)
12. Missing primary insurance status (n=611)
13. Missing metro status at the facility level (n=0)
14. Negative calculated readmission days from previous discharge (n=16)

↓

Analysis Dataset: n = 107,768

All qualified knee replacements performed from 2012Q2 through 2015Q3 (4/1/2012-9/30/2015)

Of the 4,722 excluded for race, the distribution was as follows: Asian (n=475), American Indian/Alaskan Native (n=80), Native Hawaiian/Other Pacific Islander (n=42), Two or more race groups (n=68), Other (n=2,212), Unknown (n=1,834), Missing (n=11)

© 2019 Singh JA et al. *JAMA Network Open.*

eTable 2. Demographic and Clinical Characteristics by Discharge Destination Among African-American Patients

	IRF (N=602)	SNF (N=2,997)	Home Health Care (N=2,744)	Home Self-Care (N=944)	p-value
**Sex, n (%) female**	478 (79.4)	2,407 (80.3)	1,851 (67.5)	594 (62.9)	**<0.001**
**Age, n (%)**					
<45 years	12 (2.0)	49 (1.6)	120 (4.4)	42 (4.4)	**<0.001**
45-54 years	87 (14.5)	454 (15.1)	692 (25.2)	221 (23.4)	
55-64 years	218 (36.2)	914 (30.5)	1,087 (39.6)	369 (39.1)	
65-74 years	183 (30.4)	979 (32.7)	638 (23.3)	245 (26.0)	
75-84 years	82 (13.6)	531 (17.7)	192 (7.0)	64 (6.8)	
85+ years	20 (3.3)	70 (2.3)	15 (0.5)	3 (0.3)	
**Insurance Type, n (%)**					
Medicaid	85 (14.1)	461 (15.4)	469 (17.1)	130 (13.8)	**<0.001**
Medicare / Government	347 (57.6)	1,814 (60.5)	1,097 (40.0)	350 (37.1)	
Private	170 (28.2)	722 (24.1)	1,178 (42.9)	464 (49.2)	
**Metro Area (facility level), n (%)**	592 (98.3)	2,987 (99.7)	2,708 (98.7)	937 (99.3)	**<0.001**
**Volume of Cases (by PAF and quarter), n (%)**					
<50 / quarter	138 (22.9)	639 (21.3)	472 (17.2)	117 (12.4)	0.09
50-99 / quarter	137 (22.8)	628 (21.0)	700 (25.5)	173 (18.3)	
100+ / quarter	327 (54.3)	1,730 (57.7)	1,572 (57.3)	654 (69.3)	
**Complications, n (%)**					
Venous thromboembolism (VTE)	7 (1.2)	38 (1.3)	24 (0.9)	3 (0.3)	0.14
Postoperative myocardial infarction (MI)	0 (0.0)	1 (<0.1)	0 (0.0)	0 (0.0)	n/a
Prosthetic device complication	0 (0.0)	1 (<0.1)	7 (0.3)	1 (0.1)	n/a
Surgical wound infection	1 (0.2)	0 (0.0)	3 (0.1)	0 (0.0)	n/a
**Comorbidities, n (%)**					
Congestive heart failure (CHF)	51 (8.5)	197 (6.6)	79 (2.9)	25 (2.6)	**<0.001**
Cardiac arrhythmias	86 (14.3)	418 (13.9)	249 (9.1)	72 (7.6)	**<0.001**
Valvular disease	19 (3.2)	113 (3.8)	62 (2.3)	23 (2.4)	**0.002**
Pulmonary circulation disorders	11 (1.8)	64 (2.1)	40 (1.5)	3 (0.3)	**0.002**
Peripheral vascular disorders	16 (2.7)	87 (2.9)	35 (1.3)	11 (1.2)	**<0.001**
Hypertension (combined)	442 (73.4)	2,218 (74.0)	1,936 (70.6)	650 (68.9)	**0.004**
Paralysis	9 (1.5)	12 (0.4)	3 (0.1)	1 (0.1)	**<0.001**
Other neurological disorders	26 (4.3)	118 (3.9)	64 (2.3)	19 (2.0)	**0.001**
Chronic pulmonary disease	142 (23.6)	795 (26.5)	603 (22.0)	162 (17.2)	**<0.001**
Diabetes, uncomplicated	185 (30.7)	935 (31.2)	656 (23.9)	200 (21.2)	**<0.001**
Diabetes, complicated	34 (5.6)	111 (3.7)	57 (2.1)	21 (2.2)	**<0.001**
Hypothyroidism	71 (11.8)	331 (11.0)	249 (9.1)	78 (8.3)	**0.002**
Renal failure	66 (11.0)	328 (10.9)	147 (5.4)	45 (4.8)	**<0.001**
Liver disease	13 (2.2)	77 (2.6)	60 (2.2)	17 (1.8)	0.70
Peptic ulcer disease excluding bleeding	4 (0.7)	20 (0.7)	9 (0.3)	1 (0.1)	0.07
AIDS/HIV	1 (0.2)	7 (0.2)	8 (0.3)	4 (0.4)	0.56
Lymphoma	2 (0.3)	12 (0.4)	5 (0.2)	2 (0.2)	0.60
Metastatic cancer	0 (0.0)	2 (0.1)	4 (0.1)	0 (0.0)	n/a
Solid tumor (without metastasis)	2 (0.3)	9 (0.3)	13 (0.5)	2 (0.2)	0.67
Rheumatoid arthritis/collagen vascular diseases	43 (7.1)	184 (6.1)	130 (4.7)	33 (3.5)	**<0.001**
Coagulopathy	17 (2.8)	61 (2.0)	41 (1.5)	10 (1.1)	**0.04**
Obesity	300 (49.8)	1,323 (44.1)	981 (35.8)	318 (33.7)	**0.003**
Weight loss	2 (0.3)	3 (0.1)	5 (0.2)	0 (0.0)	**<0.001**
Fluid and electrolyte disorders	64 (10.6)	279 (9.3)	183 (6.7)	46 (4.9)	**0.003**
Blood loss anemia	4 (0.7)	32 (1.1)	23 (0.8)	7 (0.7)	0.57
Deficiency anemia	24 (4.0)	95 (3.2)	83 (3.0)	19 (2.0)	0.23

© 2019 Singh JA et al. *JAMA Network Open.*

Alcohol abuse	12 (2.0)	53 (1.8)	36 (1.3)	13 (1.4)	0.26
Drug abuse	11 (1.8)	68 (2.3)	62 (2.3)	23 (2.4)	0.93
Psychoses	2 (0.3)	54 (1.8)	29 (1.1)	8 (0.8)	**0.002**
Depression	82 (13.6)	411 (13.7)	378 (13.8)	93 (9.9)	**0.007**
* Uses Wald Chi-Square from unadjusted logistic / multinomial models AND accounts for the clustering by Pennsylvania Area Facility.					

© 2019 Singh JA et al. *JAMA Network Open.*

eTable 3. Demographic and Clinical Characteristics by Discharge Destination Among White Patients

	IRF (N=7,780)	SNF (N=20,173)	Home Health Care (N=49,928)	Home Self-Care (N=22,600)	p-value
**Sex, n (%) female**	5,401 (69.4)	14,719 (73.0)	30,122 (60.3)	12,800 (56.6)	**<0.001**
**Age, n (%)**					
<45 years	40 (0.5)	79 (0.4)	812 (1.6)	439 (1.9)	**<0.001**
45-54 years	379 (4.9)	979 (4.9)	6,118 (12.3)	2,995 (13.3)	
55-64 years	1,401 (18.0)	3,623 (18.0)	17,003 (34.1)	8,287 (36.7)	
65-74 years	2,619 (33.7)	7,068 (35.0)	17,988 (36.0)	7,876 (34.8)	
75-84 years	2,459 (31.6)	7,058 (35.0)	7,428 (14.9)	2,815 (12.5)	
85+ years	882 (11.3)	1,366 (6.8)	579 (1.2)	188 (0.8)	
**Insurance Type, n (%)**					
Medicaid	156 (2.0)	490 (2.4)	1,440 (2.9)	599 (2.7)	**<0.001**
Medicare / Government	6,169 (79.3)	15,079 (74.7)	25,305 (50.7)	10,475 (46.3)	
Private	1,455 (18.7)	4,604 (22.8)	23,183 (46.4)	11,526 (51.0)	
**Metro Area (facility level), n (%)**	6,625 (85.2)	18,965 (94.0)	46,881 (93.9)	19,952 (88.3)	**0.001**
**Volume of Cases (by PAF and quarter), n (%)**					
<50 / quarter	2,174 (27.9)	3,625 (18.0)	7,574 (15.2)	2,747 (12.2)	**<0.001**
50-99 / quarter	2,276 (29.3)	5,336 (26.5)	14,263 (28.6)	5,796 (25.6)	
100+ / quarter	3,330 (42.8)	11,212 (55.6)	28,091 (56.3)	14,057 (62.2)	
**Complications, n (%)**					
Venous thromboembolism (VTE)	86 (1.1)	217 (1.1)	349 (0.7)	102 (0.5)	**<0.001**
Postoperative myocardial infarction (MI)	13 (0.2)	15 (0.1)	9 (<0.1)	4 (<0.1)	**<0.001**
Prosthetic device complication	12 (0.2)	14 (0.1)	47 (0.1)	17 (0.1)	0.24
Surgical wound infection	24 (0.3)	61 (0.3)	71 (0.1)	33 (0.1)	**<0.001**
**Comorbidities, n (%)**					
Congestive heart failure (CHF)	429 (5.5)	1,060 (5.3)	1,071 (2.1)	435 (1.9)	**<0.001**
Cardiac arrhythmias	1,392 (17.9)	3,722 (18.5)	5,583 (11.2)	2,383 (10.5)	**<0.001**
Valvular disease	456 (5.9)	1,265 (6.3)	1,758 (3.5)	659 (2.9)	**<0.001**
Pulmonary circulation disorders	168 (2.2)	412 (2.0)	482 (1.0)	134 (0.6)	**<0.001**
Peripheral vascular disorders	306 (3.9)	659 (3.3)	1,024 (2.1)	335 (1.5)	**<0.001**
Hypertension (combined)	5,273 (67.8)	13,847 (68.6)	31,640 (63.4)	13,911 (61.6)	**<0.001**
Paralysis	79 (1.0)	72 (0.4)	45 (0.1)	18 (0.1)	**<0.001**
Other neurological disorders	554 (7.1)	1,143 (5.7)	1,673 (3.4)	773 (3.4)	**<0.001**
Chronic pulmonary disease	1,493 (19.2)	3,836 (19.0)	7,344 (14.7)	3,149 (13.9)	**<0.001**
Diabetes, uncomplicated	1,948 (25.0)	4,855 (24.1)	9,095 (18.2)	3,740 (16.5)	**<0.001**
Diabetes, complicated	290 (3.7)	598 (3.0)	733 (1.5)	330 (1.5)	**<0.001**
Hypothyroidism	1,851 (23.8)	4,649 (23.0)	8,673 (17.4)	3,648 (16.1)	**<0.001**
Renal failure	720 (9.3)	1,614 (8.0)	1,822 (3.6)	756 (3.3)	**<0.001**
Liver disease	93 (1.2)	251 (1.2)	578 (1.2)	272 (1.2)	0.90
Peptic ulcer disease excluding bleeding	34 (0.4)	118 (0.6)	204 (0.4)	91 (0.4)	**0.01**
AIDS/HIV	1 (<0.1)	2 (<0.1)	4 (<0.1)	0 (<0.1)	**<0.001**
Lymphoma	22 (0.3)	72 (0.4)	100 (0.2)	39 (0.2)	**<0.001**
Metastatic cancer	10 (0.1)	22 (0.1)	24 (<0.1)	13 (0.1)	**0.002**
Solid tumor (without metastasis)	47 (0.6)	117 (0.6)	197 (0.4)	90 (0.4)	**0.002**
Rheumatoid arthritis/collagen vascular diseases	371 (4.8)	988 (4.9)	1,931 (3.9)	901 (4.0)	**<0.001**
Coagulopathy	184 (2.4)	540 (2.7)	849 (1.7)	414 (1.8)	**<0.001**
Obesity	2,293 (29.5)	5,564 (27.6)	12,681 (25.4)	5,795 (25.6)	**0.005**
Weight loss	17 (0.2)	71 (0.4)	53 (0.1)	15 (0.1)	**<0.001**
Fluid and electrolyte disorders	806 (10.4)	2,297 (11.4)	3,561 (7.1)	1,664 (7.4)	**<0.001**
Blood loss anemia	100 (1.3)	259 (1.3)	441 (0.9)	123 (0.5)	**<0.001**
Deficiency anemia	257 (3.3)	507 (2.5)	789 (1.6)	315 (1.4)	**<0.001**

© 2019 Singh JA et al. *JAMA Network Open.*

Alcohol abuse	55 (0.7)	215 (1.1)	444 (0.9)	190 (0.8)	0.13
Drug abuse	77 (1.0)	194 (1.0)	321 (0.6)	117 (0.5)	**<0.001**
Psychoses	84 (1.1)	204 (1.0)	137 (0.3)	56 (0.2)	**<0.001**
Depression	1,446 (18.6)	3,934 (19.5)	8,086 (16.2)	3,396 (15.0)	**<0.001**
* Uses Wald Chi-Square from unadjusted logistic / multinomial models AND accounts for the clustering by Pennsylvania Area Facility.					

© 2019 Singh JA et al. *JAMA Network Open.*

eFigure 2. Adjusted Association of Race/Ethnicity With 90-Day Hospital Readmission

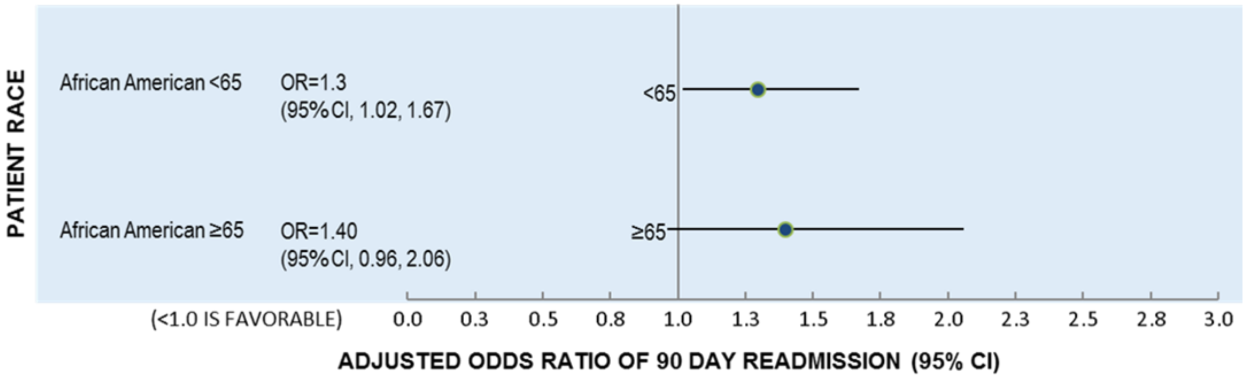

X-axis shows the adjusted odds ratios [OR] of the association of race with 90-day hospital readmission after knee replacement surgery in African-Americans compared to white patients (reference category); the Y-axis shows each of the two age categories of African-Americans (<65 and ≥65 year groups).

The point estimate is shown with a solid circle and the 95% confidence intervals with horizontal lines. A vertical line for adjusted odds ratio passes through 1.0, indicating no statistical significance. Compared to white, African-American race was not associated with the risk of 90-day hospital readmission in ≥65 year group, but was significantly associated with higher odds of 90-day hospital readmission in <65 age group.

Variables adjusted in the model:

For these models with 90-day readmission as outcome, we included all of the potential covariates (age, gender, insurance type, comorbidities, metro area location, hospital annual TKA volume, complications) from the corresponding descriptive tables except for the following that had a p-value >0.10:

(1) <u><65 years</u>: Female (p=0.11), metro area location (p=0.50), volume of cases by quarter (p=0.25), postop MI (p=0.16), valvular disease (p=0.52), hypothyroid (p=0.77), peptic ulcer disease (p=0.72)

(2) ≥65 <u>years</u>: volume of cases by quarter (p=0.98), prosthetic device complication (p=0.89), hypertension (p=0.13), hypothyroid (p=0.36), solid tumor without metastasis (p=0.36)

© 2019 Singh JA et al. *JAMA Network Open.*
